# Wasting condition as a marker for severe disease in pediatric Crohn's disease

**DOI:** 10.1097/MD.0000000000029296

**Published:** 2022-05-27

**Authors:** Wook Jin, Dong-Hwa Yang, Hann Tchah, Kwang-An Kwon, Jung-Ho Kim, Su-Jin Jeong, Ki-Baik Hahm

**Affiliations:** a Department of Pediatrics, Gachon University Gil Medical Center, Incheon, Korea; b Department of Gastroenterology, Gachon University Gil Medical Center, Incheon, Korea; c CHA University Bundang Medical Center Digestive Disease Center, Seongnam, Korea; d Medpacto Research Institute, Medpacto, Seoul, Korea.

**Keywords:** crohn's disease, disease marker, pediatric, sarcopenia, wasting syndrome

## Abstract

Several studies have shown an association between sarcopenia and clinical outcomes in patients with Crohn's disease (CD). However, studies have shown different results, and the association between prognosis and wasting conditions in pediatric patients with CD is uncertain. In this study, we evaluated the clinical significance of wasting in pediatric CD patients.

We retrospectively analyzed data on wasting syndrome in patients diagnosed with CD at the Pediatric Department of Gachon University Gil Medical Center between January 1995 and January 2018.

Of 105 patients diagnosed with CD, 39.0% were classified into the wasting group (weight-for-age z-score ≤−1) and 61.0% into the nonwasting group (weight-for-age z-score >−1). Height-for-age and body mass index-for-age z-scores at the time of diagnosis were significantly associated with wasting (*P* < .001 and *P* < .001, respectively). Additionally, wasting was significantly associated with low levels of hemoglobin (*P* < .001), high levels of inflammatory markers, including C-reactive protein (*P* = .005) and erythrocyte sedimentation rate (*P* = .04), and a smaller surface area of the gluteus maximus muscle (*P* < .001). Interestingly, since the site of CD involvement and other markers for nutrition did not correlate with wasting syndrome, wasting appears to be a marker for the severity of pediatric CD. Lastly, the wasting group tended to have a greater use of biologic therapy after first-line therapy failed to improve wasting syndrome.

Wasting syndrome, including sarcopenia, can serve as a marker for the severity of pediatric CD.

## 1 Introduction

Crohn's disease (CD) is a chronic inflammatory bowel disease that usually develops in adolescence and early adulthood.^[1]^ Pediatric CD is characterized by malabsorption, wasting condition referring to weight loss, growth failure, nutritional deficiencies, malnutrition, low body mass index (BMI), and sarcopenia, in addition to the classical symptoms of abdominal pain and diarrhea.^[2–4]^ Therefore, CD-associated undernutrition is common in pediatric patients with CD. Weight loss associated with sarcopenia remains a cornerstone for the diagnosis of CD.^[5]^ Children with CD have features of nutritional cachexia with normal fat stores but depleted lean mass. They present with a lower concentration of plasma micronutrients, anemia, and alterations in trace elements. However, changes in nutrition are not sufficient to explain sarcopenia because antagonizing the sarcopenic TNF-α antibody with infliximab can improve sarcopenia.^[6]^ Patients of different sexes with CD differ in body composition,^[7]^ and improvement of lean body mass can be achieved with physical activity and anti-inflammatory medicines.^[8]^

Although the exact mechanism of CD-related sarcopenia is currently unknown, repeated intestinal inflammation, disuse, or debility, and glucocorticoid therapy may play a role.^[9,10]^ Several possible explanations for sarcopenia in CD have been suggested. First, the inflammatory process, in particular, high levels of sarcopenic inflammatory mediators, such as TNF-α and IL-6, are believed to be related to increased muscle-specific protein degradation and decreased muscle synthesis.^[11–13]^ Second, patients with CD seem to be unable to maintain a positive postprandial protein balance^[14]^ and could be deficient in vitamin D.^[15]^ and growth hormone.^[16]^ Lastly, there may be alterations in the intestinal microbiota.^[17–20]^ The etiology of CD is believed to be multifactorial, including changes in the intestinal microbiota. Dysbiosis in CD comprises a decrease in *Bacteroides* and *Firmicutes* bacteria and an increase in *Gammaproteobacteria* and *Actinobacteria*. As a result of this increase, bacteria cross the mucosal barrier, attach to the intestinal epithelial cells, and replicate within the macrophages, stimulating the secretion of TNF-α.^[17]^ The significance of the interplay between gut microbiota and other organs has recently emerged.^[18]^

Based on studies of wasting syndrome, including sarcopenia in cachexia models that were associated with gut barrier dysfunction, intestinal polyposis, inflammatory responses in APC/^Min+^ mice,^[21]^ and prominent manifestations of pediatric CD, we hypothesized that wasting syndrome could be a marker of severe systemic disease in pediatric CD. Therefore, we evaluated the significance of wasting syndrome in pediatric patients with CD.

## 2 Patients and methods

### 2.1 Study population and data collection

Patients aged <18 years who were diagnosed with CD at Gachon University Gil Medical Center (Incheon, Korea) between July 1995 and January 2018 were eligible for the study. Patients whose diagnoses were confirmed by primary colonoscopy, biopsy, or other investigations were included. Patients for whom CD could not be confirmed with colonoscopy and those who had already been diagnosed and treated for CD at another hospital were excluded. The study complied with the principles of the Declaration of Helsinki. The study protocol was reviewed and approved by the Institutional Review Board of the Gachon University College of Medicine (GCIRB 2018-202).

CD diagnosis was based on conventional clinical, radiologic, endoscopic, and histopathological criteria.^[22]^ Data collected from medical records included age at diagnosis, sex, height, weight, laboratory findings, medication use, colonoscopy results, and abdominopelvic computed tomography (CT) results. BMI was calculated as weight divided by height in meters squared (kg/m^2^). The use of mesalamine, azathioprine, infliximab, and adalimumab were documented. Our center only prescribed biologics as a step-up strategy when primary therapy failed. We investigated the use of medication based on wasting syndrome and whether a top-down strategy was required.

### 2.2 Definition of wasting condition

Sex-specific height-for-age z-score (HAZ), weight-for-age z-score (WAZ), and BMI-for-age z-score (BMIZ) were calculated using the LMS method. Patients were divided into the following 2 groups based on their WAZ: patients with a WAZ ≤−1 were classified into the wasting group and those with a WAZ >−1 were classified into the nonwasting group. The standard deviation scores (z-scores) were used to evaluate and compare anthropometric measurements among children of various ages and sexes. The z-score, which is a standardized value based on data from a normal pediatric population, is calculated using values appropriate for the child's age and sex. The HAZ, WAZ, and BMIZ for each patient were calculated to 2 decimal places after accounting for age and sex.

### 2.3 Laboratory studies

Venous blood samples were obtained at the time of the diagnosis. Complete blood cell count, serum albumin (g/dL), C-reactive protein (CRP) (mg/dL), erythrocyte sedimentation rate (ESR) (mm/h), calprotectin (μg/mg), and micronutrients such as 25-OH vitamin D were measured using standard techniques. The association between wasting syndrome and laboratory findings at diagnosis was also investigated.

### 2.4 Colonoscopy and abdominopelvic computed tomography image

**Figure 1:**
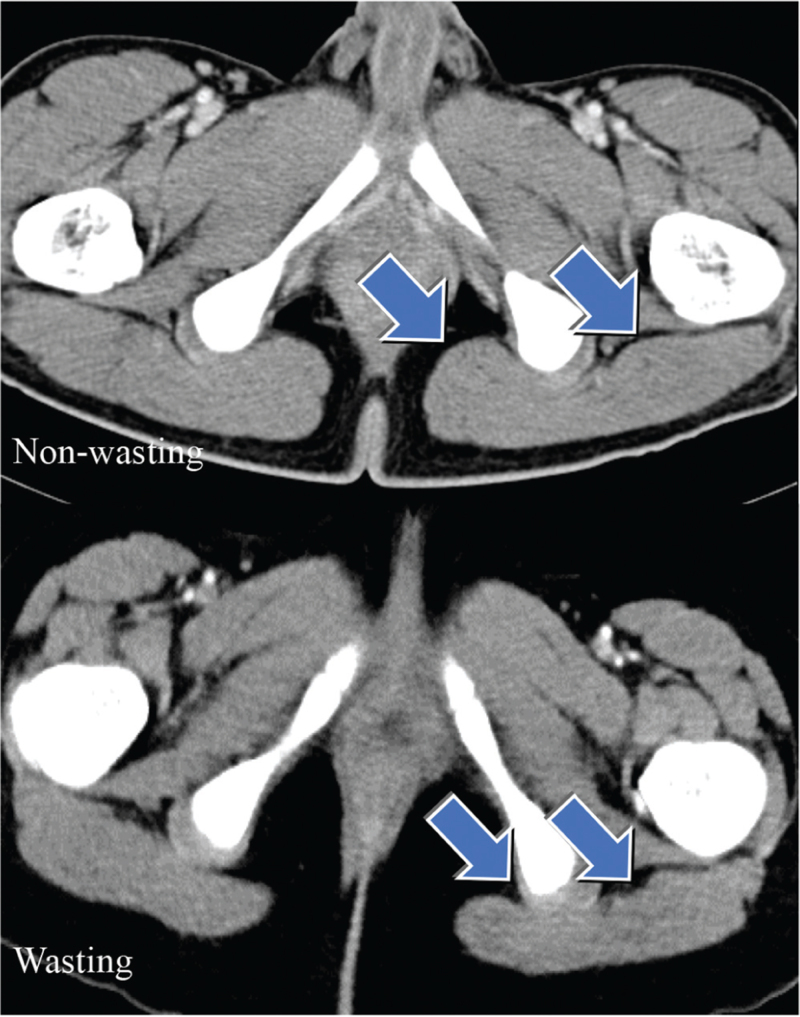
Left gluteus maximus muscle (arrow) surface areas of the patients on a single image of computed tomography at the most thickened level of the left ischiofemoral space.

Colonoscopy was performed, and the locations of the lesions (the ileum, colon, and anus) as well as the extent of organ involvement were documented. Images were obtained from digital storage using image-viewing software. CT image analysis was performed using the INFINITY PACS software. The left *gluteus maximus* muscle surface area (cm^2^) was measured on a single image at the thickest section of the left ischiofemoral space (Fig. 1). This surface area was then compared between the wasting and non-wasting groups. Muscle masses in children, unlike in adults, vary by age and sex. Therefore, the left *gluteus maximus* muscle surface area z-scores were calculated according to age and sex. The mean and standard deviation of the left *gluteus maximus* muscle surface area of 30 normal control patients for each age and sex were calculated. The surface area, measured as mentioned above, was converted to the muscle surface area z-scores.

### 2.5 Statistical analyses

Analyses were performed using MedCalc *Ver.* 18.2.1 (MedCalc Software bvba, Ostend, Belgium). Student *t* test or the Mann–Whitney *U* test was used to test the associations between continuous variables, and the Chi-Squared test or Fisher exact test was used to test the associations between categorical variables. Statistical significance was set at *P* < .05. Univariate regression was used to evaluate the association between each variable and wasting. Multivariate regression was used to evaluate the association between wasting and selected variables while controlling for confounders.

## 3 Results

**Figure 2:**
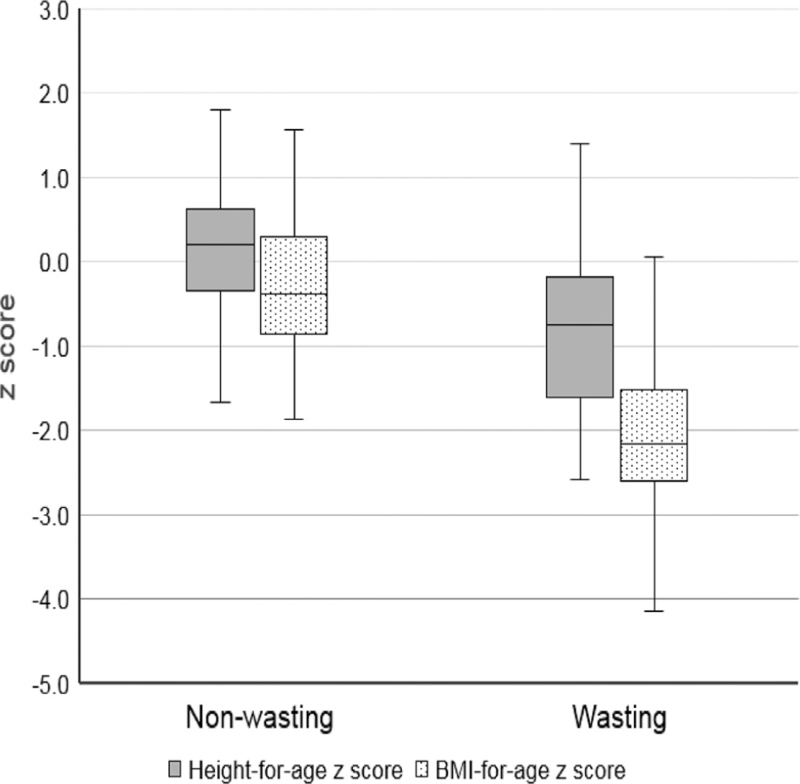
Box plots demonstrating the association between wasting conditions and lower height-for-age and BMI-for-age z-scores.

Among the 494 patients who visited our hospital and were suspected to have CD, 105 patients were included in this study after confirmation of CD by colonoscopy, biopsy, and laboratory investigation. As shown in Table 1, among the 105 patients confirmed with CD, 41 and 64 patients were classified into the wasting and nonwasting group, respectively. The participants’ characteristics are presented in Table 1. There were no significant differences in age or sex distributions between the 2 groups. However, BMI, height-for-age, and BMIZ were all significantly lower in participants with wasting than in those without wasting (*P* *<* .001) (Table 1; Fig. 2). Initial laboratory reports showed that hemoglobin, hematocrit, and albumin levels were significantly lower in the wasting group than in the nonwasting group (*P* *<* .001) (Table 2); however, these levels were not abnormal. Furthermore, the mean CRP and ESR levels were significantly higher in the wasting group than in the nonwasting group (*P* *<* .05) (Table 2). These data suggest that wasting syndrome is associated with increased inflammatory responses. There was no significant difference in the levels of calprotectin, antineutrophil cytoplasmic antibodies, and anti-Saccharomyces cerevisiae antibodies. We compared micronutrients, including 25-OH vitamin D, vitamin B12, vitamin E, zinc, and selenium, between the wasting and nonwasting groups; however, no significant differences were found (Table 3). We wondered whether the site of involvement and the extent of involvement were associated with wasting syndrome. There was no difference in disease location, except for a significantly higher involvement of the anus in the nonwasting group than in the wasting group (*P* *<* .05) (Table 4).

**Table 1:** Age, z-score, and body mass index according to wasting conditions.

	Non-wasting group (n = 64)	Wasting group (n = 41)	*P* value
Age, years^∗^	15.1 ± 2.8	15.6 ± 1.8	.28
Male, n (%)	48 (75.0)	27 (65.9)	.38
BMI, kg/m^2^^†^	19.6 (18.3–21.9)	16.0 (14.9–17.4)	<.001
Height-for-age z-score^†^	0.20 (−0.35–0.62)	−0.75 (−1.6–-0.19)	<.001
BMI-for-age z-score^†^	−0.22 ± 0.80	−2.18 ± 1.13	<.001

BMI = body mass index.

∗ Data are expressed as mean (standard deviation).

† Data are expressed as median (interquartile range).

**Table 2:** Core laboratory findings according to wasting conditions.

	Non-wasting group (n = 64)	Wasting group (n = 41)	*P* value
Hemoglobin (g/dL)^∗^	12.5 ± 2.0	11.3 ± 1.3	<.001
Hematocrit (%)^∗^	38.0 ± 5.0	35.1 ± 2.9	<.001
WBC (/μL)^†^	8465 (6450–11593)	8700 (6870–11420)	.79
Platelet (10^9^/L)^†^	349 (280–473)	441 (365–545)	.004
Albumin (g/dL)^†^	4.2 (3.8–4.4)	3.6 (3.2–4.0)	<.001
Prealbumin (mg/dL)^†^	18.9 (14.9–22.2)	13.3 (10.3–17.1)	.08
CRP (mg/dL)^†^	1.63 (0.11–5.92)	5.95 (2.59–10.6)	.005
ESR (mm/h)^†^	23.5 (6.3–42.8)	43.0 (22.5–59.5)	.041
Calprotectin (mg/kg)^†^	988 (207–1412)	1998 (691–1672)	.22
ANCA +, n (%)	3 (6.0)	2 (5.7)	1.0
ASCA +, n (%)	10 (30.3)	11 (47.8)	.26

ANCA = antineutrophil cytoplasmic antibodies, ASCA = anti-Saccharomyces cerevisiae antibodies, CRP = C-reactive protein, ESR = erythrocyte sedimentation rate, WBC = white blood cell.

∗ Data are expressed as mean (standard deviation).

† Data are expressed as median (interquartile range).

**Table 3:** Laboratory findings of minor nutrient parameters according to wasting conditions.

	Nonwasting group (n = 64)	Wasting group (n = 41)	*P* value
25-OH Vitamin D (ng/mL)^∗^	15.0 (8.8–19.5)	10.7 (4.5–12.1)	.06
Vitamin B12 (pg/mL)^∗^	595 (460–751)	508 (356–866)	.59
Vitamin E (μmol/L)^∗^	17.7 (15.2–20.4)	18.8 (16.0–22.3)	.69
Zinc (μg/dL)^∗^	87.0 (73.5–96.5)	78.0 (60.0–87.0)	.17
Selenium (μg/L)^∗^	99.0 (90.8–104.3)	93.0 (86.5–97.5)	.17

∗ Data are expressed as median (interquartile range).

**Table 4:** Disease location according to wasting conditions.

	Nonwasting group (n = 60)	Wasting group (n = 36)	*P* value
Ileum, n (%)	15 (25.0)	6 (16.7)	.34
Ileo-colic, n (%)	30 (50.0)	24 (63.9)	.11
Colon, n (%)	15 (25.0)	7 (19.4)	.53
Anal involvement, n (%)	21 (32.8)	6 (14.6)	.038

**Figure 3:**
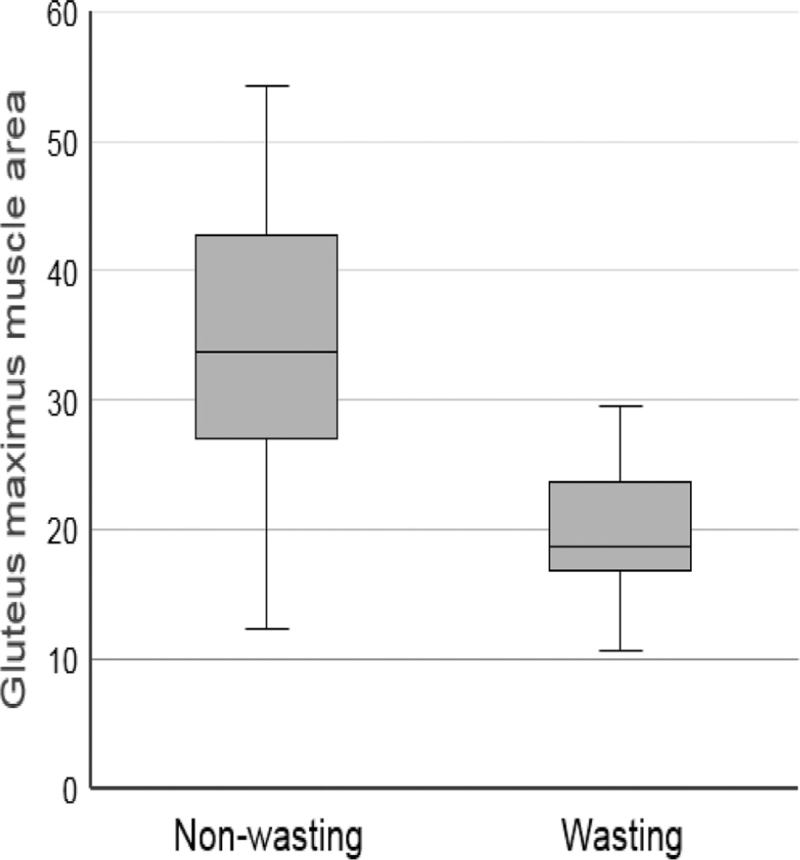
Box plot demonstrating that wasting conditions are associated with small left gluteus maximus muscle surface areas.

Muscle mass, measured on a single CT image as the surface area of the *gluteus maximus* (Fig. 1), was compared between the 2 groups. Compared with the nonwasting group, the wasting group had significantly lower muscle surface area (33.8 vs 18.7 cm^2^, *P* *<* .001) (Fig. 3). Since these muscle masses were analyzed objectively after the z-score, both the nonwasting and wasting groups had negative muscle mass z-scores. However, the z-scores of muscle mass in the wasting group were significantly lower than those in the non-wasting group (*P* *<* .001) (Table 5).

**Table 5:** Comparative muscle mass z-scores in the non-wasting and wasting groups in pediatric CD.

	Nonwasting group	Wasting group	*P* value
Muscle mass z-score	−1.2 (−2.1–−0.3)	−2.9 (−3.5–−2.3)	<.001

Data are expressed as median (interquartile range). CD = Crohn's disease.

Finally, in the univariate analyses, the presence of lean mass deficit, CRP, and ESR predicted wasting syndrome (*P* *<* .05). However, in the multivariable analysis, only lean mass deficit and CRP were significant predictors of wasting syndrome (*P* *<* .05) (Table 6). Since wasting syndrome was significantly correlated with inflammatory markers, we assessed the therapeutic approach in each group. The wasting group received more interventions with biologicals than the nonwasting group (*P* *<* .01) (Table 7).

**Table 6:** Univariate and multivariate analyses using logistic regression with parameters implicated in wasting conditions.

	Univariate		Multivariate	
	OR (95% CI)	*P* value	OR (95% CI)	*P* value
CRP	1.190 (1.089–1.299)	.001	1.26 (0.999–1.588)	.04
ESR	1.016 (1.001–1.031)	.03	0.971 (0.935–1.008)	.12
Muscle mass z-score	0.046 (0.009–0.221)	<.001	0.04 (0.006–0.263)	.01

CI = confidence interval, CRP = C-reactive protein, ESR = erythrocyte sedimentation rate, OR = odds ratio.

**Table 7:** Medications used in the wasting and nonwasting groups.

Variable	Nonwasting group (n = 64)	Wasting group (n = 41)	*P* value
Mesalazine, n (%)	61 (95.3)	38 (92.7)	.68
Azathioprine, n (%)	10 (15.6)	7 (17.1)	.84
Biologics, n (%)	17 (26.6)	21 (51.2)	.01
Infliximab (%)	10 (15.6)	12 (29.3)	.09
Adalimumab (%)	7 (10.9)	9 (21.9)	.13

## 4 Discussion

In this study, we found that sarcopenia was associated with low height-for-age, low BMI-for-age, high levels of inflammatory markers such as CRP and ESR, and low levels of hemoglobin, hematocrit, and albumin in 105 children diagnosed with primary CD. Since there was no association between wasting syndrome and disease location or nutritional derangement, malabsorption was not the main cause of wasting. Fewer patients with wasting responded to primary therapy and received biological therapy. We concluded that in addition to current markers for assessing CD severity, clinical evaluation of sarcopenia should be mandatory for pediatric CD.

Some studies have shown an association between altered body composition and clinical outcomes in patients with CD. In adults, Zhang et al^[23]^ reported an association between decreased lean mass and postoperative complications. In children, Thayu et al^[7]^ did not find an association between body composition and disease activity, but they did show a correlation between body composition and inflammatory markers. Another study by Burnham et al^[8]^ showed that lean mass deficit was positively correlated with disease activity. Conclusively, altered body composition might reflect “inflammatory activities” in inflammatory bowel diseases (IBD). However, sarcopenia is a manifestation of altered body composition, and our study suggests that sarcopenia is associated with increased disease activity in pediatric CD, independent of nutritional derangement. A major difference between our study and previous studies is that patients were divided into 2 groups based on weight-for-age z-scores. For adults, wasting, especially lean mass deficit, is well-recognized for its clinical significance in many diseases.^[20,24–27]^ These data suggest that wasting syndrome, deficit, and lean mass can indicate poor prognosis in pediatric CD, and more efforts to restore weight should be considered for patients with wasting syndrome.

Extraintestinal manifestations in children with IBD, growth retardation, aphthous ulcers, arthropathy, oculopathy, and skin lesions are common, and all of them are associated with immune-mediated pathogenesis.^[28,29]^ Since malnutrition and growth retardation are important issues in pediatric CD, Song et al^[30]^ investigated the prevalence of nutritional and growth status in Korean children with CD and found subnormal serum levels of hemoglobin, albumin, iron, ferritin, calcium, magnesium, folate, vitamin B12, and zinc. However, in our study (Tables 2 and 3), these derangements were due to disease characteristics. We did not assess growth retardation and nutritional derangement in pediatric CD, but our study clearly leads to the novel conclusion that sarcopenia should be considered as a core manifestation of CD. Growth retardation, usually present in 6.9% to 15% of children with CD,^[31]^ results from severe malnutrition. In contrast, sarcopenia results from severe inflammatory insult in pediatric CD, necessitating intensive anti-inflammatory agents, including biologicals.

In an animal cachexia model, treatment with BPC157 (body protection compound 157) with TNF-α antagonists, such as infliximab or adalimumab, not only improved cachexia but also improved the prognosis of cancer.^[32]^ Subramaniam et al^[6]^ reported that infliximab reverses inflammatory sarcopenia in CD, and Dos Santos et al^[33]^ reported that all components of body composition improved after therapy with infliximab. Therefore, for patients with wasting syndrome, early introduction of biological treatments such as infliximab and adalimumab may be needed to improve prognosis. In reality, sustained inflammation, steroid treatment, and malnutrition contribute to decreased growth rate and increased cytokine production, and growth hormone insensitivity might be a major mechanism involved in either growth retardation or sarcopenia.^[34]^ Reduction of inflammation through accelerated top-down treatment using biologics can be the cornerstone of treatment, and restoring sarcopenia with these modalities might be a successful therapeutic plan, although more research is required.

A potential limitation of this study is the lack of consideration of the Tanner stage. In the case of pediatric CD, the Tanner stage has profound effects on height, weight, and body composition such as lean mass, especially in adolescents. Magnetic resonance imaging has recently been developed to offer 3-dimensional volume calculation to allow for calculation of muscle volume.^[35,36]^ However, to measure lean mass, only CT imaging data were used in our study. In addition to radiologic data, bioelectrical impedance, isotope dilution, total body potassium, and dual-energy X-ray absorptiometry can be used to measure body composition.^[37]^ Some researchers argue that a high resolution CT is required. We included pediatric patients who required evaluation of the gastrointestinal conditions in whom sarcopenia was evaluated. Therefore, assessment of sarcopenia can be performed with other types of evaluation in pediatric patients. Another limitation of our study is the small sample size; the study was performed at a single medical center study, and it was a retrospective analysis. The small sample size can be explained by the fact that we only included cases diagnosed with primary CD and excluded referral cases. It is worth mentioning that our institute is one of the biggest medical centers in Korea. Lastly, because the study was retrospective, we were not able to obtain additional data for disease severity, such as the PCDAI score from the early participants. For the prospective study, additional data on disease severity should be included.

In conclusion, we analyzed cases according to z-scores (including HAZ, WAZ, and BMIZ) reflecting objective measurements. The characteristics of wasting syndrome can be summarized as having low HAZ and BMIZ, high levels of inflammatory markers, a deficit in lean mass, and low response rates to primary treatment other than biologicals. Therefore, wasting syndrome, including sarcopenia, can be a marker of the severity of pediatric CD.

## Author contributions

**Conceptualization:** Hann Tchah, Jung-Ho Kim, Ki-Baik Hahm, Kwang-An Kwon, Su-Jin Jeong.

**Data curation:** Dong-Hwa Yang, Hann Tchah, Ki-Baik Hahm.

**Formal analysis:** Dong-Hwa Yang, Wook Jin.

**Investigation:** Dong-Hwa Yang, Wook Jin.

**Supervision:** Hann Tchah, Ki-Baik Hahm.

**Writing – original draft:** Wook Jin.

**Writing – review & editing:** Wook Jin.
